# Detection of *Chlamydiaceae* and *Chlamydia*-like organisms on the ocular surface of children and adults from a trachoma-endemic region

**DOI:** 10.1038/s41598-018-23887-1

**Published:** 2018-05-09

**Authors:** Ehsan Ghasemian, Aleksandra Inic-Kanada, Astrid Collingro, Florian Tagini, Elisabeth Stein, Hadeel Alchalabi, Nadine Schuerer, Darja Keše, Balgesa Elkheir Babiker, Nicole Borel, Gilbert Greub, Talin Barisani-Asenbauer

**Affiliations:** 1 OCUVAC – Center of Ocular Inflammation and Infection, Laura Bassi Centres of Expertise, Center for Pathophysiology, Infectiology and Immunology, Medical University of Vienna, Vienna, Austria; 2 Department of Microbiology and Ecosystem Science, Division of Microbial Ecology, University of Vienna, Vienna, Austria; 3 Institute of Microbiology, University of Lausanne and University Hospital, Lausanne, Switzerland; 4 Institute of Microbiology and Immunology, Faculty of Medicine, University of Ljubljana, Ljubljana, Slovenia; 5 Federal Ministry of Health, Khartoum, Sudan; 6 Institute of Veterinary Pathology, Department of Pathobiology, Vetsuisse Faculty, University of Zurich, Zurich, CH-8057 Switzerland; 7 Infectious Diseases Service, University Hospital of Lausanne, Lausanne, Switzerland

## Abstract

Trachoma, the leading infectious cause of blindness, is caused by *Chlamydia trachomatis* (Ct), a bacterium of the phylum *Chlamydiae*. Recent investigations revealed the existence of additional families within the phylum *Chlamydiae*, also termed *Chlamydia*-like organisms (CLOs). In this study, the frequency of Ct and CLOs was examined in the eyes of healthy Sudanese (control) participants and those with trachoma (case). We tested 96 children (54 cases and 42 controls) and 93 adults (51 cases and 42 controls) using broad-range *Chlamydiae* and Ct-specific (*omcB*) real-time PCR. Samples positive by broad-range *Chlamydiae* testing were subjected to DNA sequencing. Overall *Chlamydiae* prevalence was 36%. Sequences corresponded to unclassified and classified *Chlamydiae*. Ct infection rate was significantly higher in children (31.5%) compared to adults (0%) with trachoma (p < 0.0001). In general, 21.5% of adults and 4.2% of children tested positive for CLOs (p = 0.0003). Our findings are consistent with previous investigations describing the central role of Ct in trachoma among children. This is the first study examining human eyes for the presence of CLOs. We found an age-dependent distribution of CLO DNA in human eyes with significantly higher positivity in adults. Further studies are needed to understand the impact of CLOs in trachoma pathogenicity and/or protection.

## Introduction

Trachoma constitutes the leading infectious cause of blindness worldwide^1^. It is considered a public health problem in 41 countries and is responsible for the visual impairment of approximately 1.9 million people, of whom 0.450 million are blind^2^. Trachoma is caused by repeated infection of the conjunctiva with *Chlamydia trachomatis*, a bacterium of the phylum *Chlamydiae*^1,3^. The majority of infections heal without sequelae, whereas subgroups of infected patients develop chronic inflammation and scarring of the ocular surface. However, the factors responsible for the different outcomes are not yet fully understood.

The phylum *Chlamydiae* was originally considered to consist of only a single family, the *Chlamydiaceae*^4^. Since the mid-1990s, advances in molecular techniques have uncovered new *Chlamydiae*, termed *Chlamydia*-like organisms (CLOs) or environmental *Chlamydiae*, belonging to novel families in this phylum; i.e., the *Parachlamydiaceae*, *Simkaniaceae*, *Rhabdochlamydiaceae*, *Waddliaceae*, *Candidatus* Piscichlamydiaceae, *Candidatus* Parilichlamydiaceae, *Candidatus* Amphibiichlamydiaceae, *Candidatus* Clavichlamydiaceae, and *Criblamydiaceae*^5–9^. There is also molecular evidence for an even greater diversity within the phylum, suggesting the existence of more than 180 families therein^10^. *Chlamydiae* are obligate intracellular bacteria with a biphasic developmental cycle that includes an extra-cellular elementary body and an intracellular dividing reticulate body^11–13^. CLOs have been identified from various environmental sources and hosts such as humans, warm-blooded terrestrial vertebrates, fish, reptiles, amphibians, arthropods, and eukaryotic microorganisms such as amoebae^6–9,14,15^. *Parachlamydia acanthamoebae* and *Neochlamydia hartmannellae* were found in eyes of cats with keratitis, conjunctivitis, and other ocular diseases^16,17^. In a study on the eyes of sheep with conjunctivitis, DNA of uncultured CLOs was found in two-thirds (26/32) of the samples^18^. The presence of *P*. *acanthamoebae* and other CLOs have also been reported in symptomatic eyes of guinea pigs and in the one-day disposal contact lenses of their owner^19^. Additionally, results from several studies on humans and animals have shown the presence of CLOs in urogenital sites, the skin, respiratory tract and eye^20–22^. Furthermore, to date, *Parachlamydiaceae* species (spp.), *Simkania negevensis* and *Waddlia chondrophila* have been linked to human respiratory diseases and adverse pregnancy outcomes with various levels of evidence^5,23–27^.

Increasing evidence suggests an association between the presence of non-*C*. *trachomatis* bacteria and trachoma. Dean *et al*. reported a high prevalence of *Chlamydia psittaci*, *Chlamydia suis*, *Chlamydia pecorum*, and *Chlamydia pneumoniae* in eyes of patients with trachoma from Nepal^28,29^. Results from two separate studies in Tanzania, one, a cross-sectional study on children^30^ and the other a case-control study on adults^31^, revealed a strong association between non-chlamydial bacterial infections (such as *Streptococcus pneumoniae* and *Haemophilus influenza*) and clinical signs of trachoma in children and adults. These findings were independently supported by two studies on Gambian children and adults with signs of trachoma^32,33^. In addition, a study in Ethiopia suggested that conjunctival colonization with pathogenic bacterial species is more likely in patients with trachomatous trichiasis^34^. Trichiasis, the introversion of the eyelashes, has been described repeatedly as a direct route for conjunctival colonization with pathogens in the eyes of patients with trachoma^34,35^.

**Figure 1:**
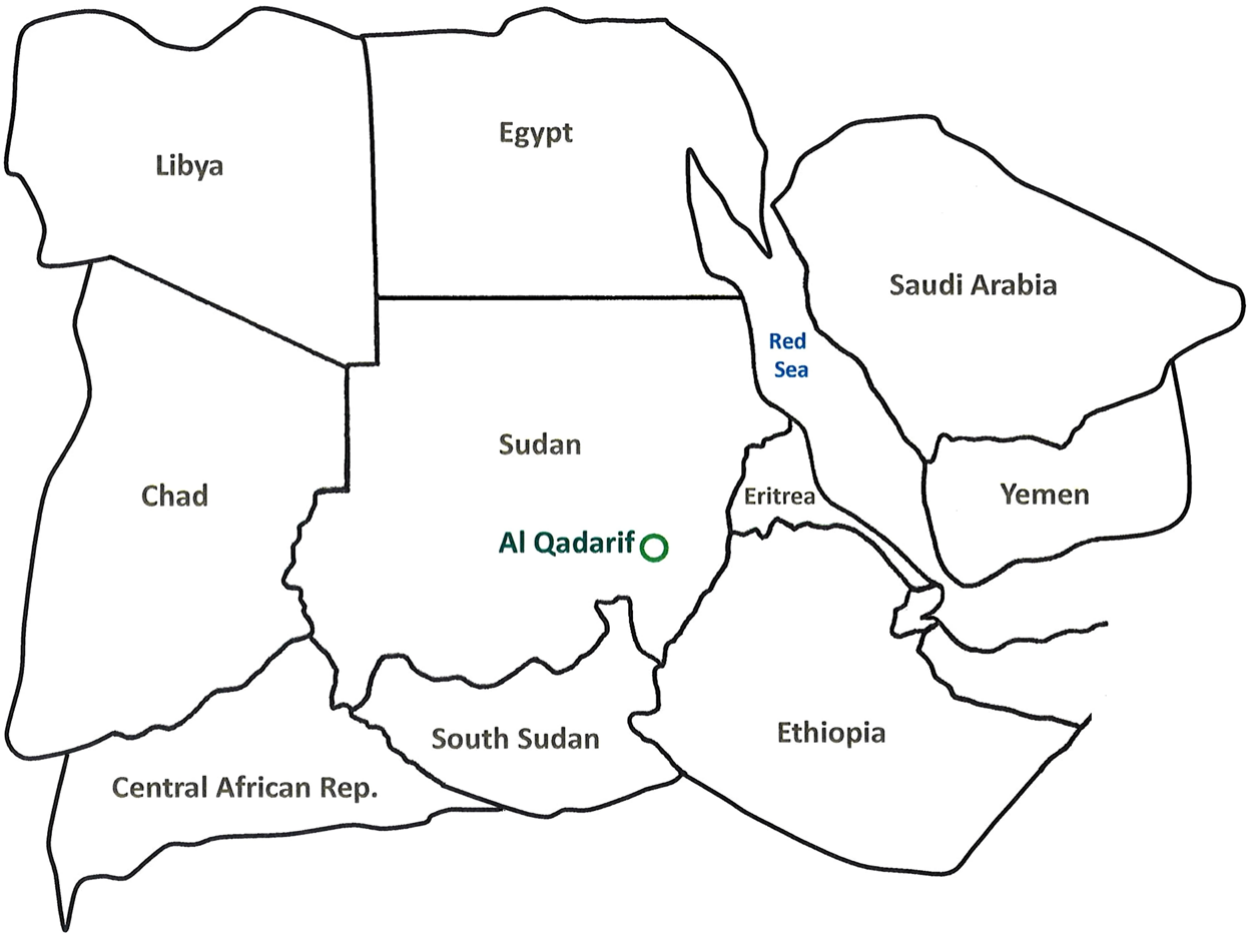
Location of sampling sites:  Al Qadarif region, Sudan. The Map marks the city of Al Qadarif, which is the capital of the state Al Qadarif in central Sudan (the map is generated in Microsoft PowerPoint 2016 Software).

Altogether, such surveys provide insights into the possible role of various chlamydial species on ocular surface pathogenicity. Several similarities have been demonstrated between different CLOs and the *Chlamydiaceae* concerning their developmental cycle, virulence factors, and invasion mechanisms to the host cells^5,10,20,36,37^. Considering the importance of *C*. *trachomatis* in trachoma and the status of CLOs as highly prevalent microorganisms in the environment, it is necessary to examine the frequency of CLOs on the ocular surface and their possible association with trachoma. The main aim of this case-control study was therefore to investigate the frequency of *C*. *trachomatis* and other *Chlamydiae* in ocular samples of children and adults from Al Qadarif region in Sudan (Fig. 1) with trachoma and healthy participants.

## Results

### Study population

The distribution of participants by age, sex, and grade of trachoma is shown in Table 1. The study population comprised 189 individuals, of whom 96 (children) were aged 1–9 years and 93 (adults) aged 15–88 years. The median ages of the children and adults were 5 and 56.3 years, respectively.

**Table 1:** Baseline and clinical characteristics of study participants in Al Qadarif-Sudan.

	Children case (N = 54)		Children control (N = 42)		Adults case (N = 51)		Adults control (N = 42)	
	n	%	n	%	n	%	n	%
**Sex**								
Male	31	57.4	22	52.4	17	33.3	12	28.6
Female	23	42.6	20	47.6	34	66.7	30	71.4
**Age**								
9≤	54	100	42	100	—	—	—	—
15–25	—	—	—	—	2	3.9	2	4.8
26–35	—	—	—	—	4	7.8	4	9.5
36–45	—	—	—	—	8	15.7	8	19
46–55	—	—	—	—	7	13.7	8	19
56–65	—	—	—	—	11	21.6	4	9.5
>65	—	—	—	—	19	37.2	16	38.1
**Trachoma grade**								
TF	54	100	0	0	—	—	0	0
TI	—	—	0	0	—	—	0	0
TS	—	—	0	0	—	—	0	0
TT	—	—	0	0	51	100	0	0

### Broad-range Chlamydiae real-time polymerase chain reaction and sequence classification

**Figure 2:**
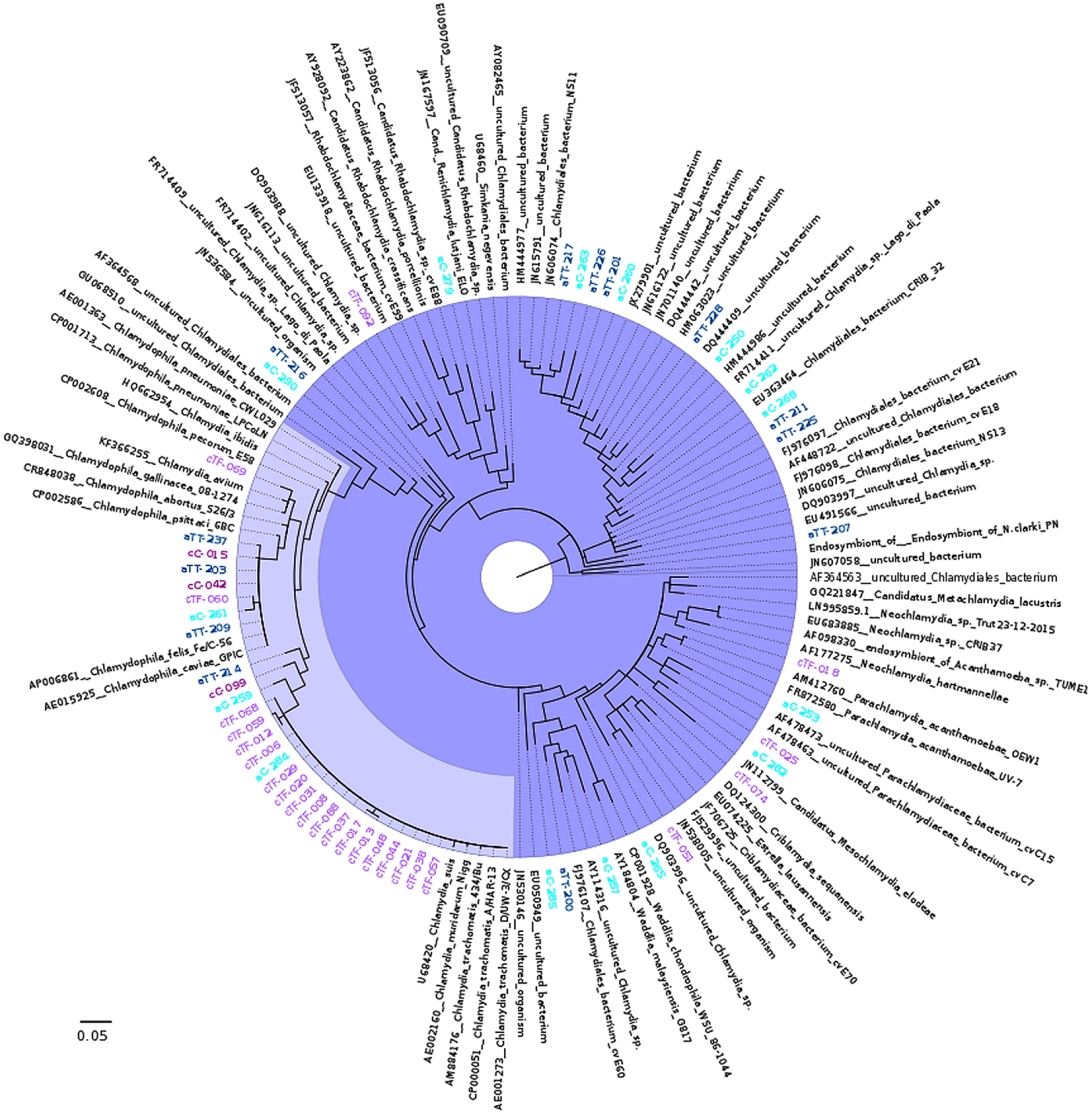
Phylogenetic affiliation of 16S rRNA gene sequences detected in this study. A Bayesian tree built from full-length chlamydial sequences is shown, to which the partial sequences generated in this study were added using Parsimony. Only sequences closely related to the sequences found in this study are depicted. The full dendrogram including all used sequences is available as Supplementary Fig. S2. Branches belonging to the *Chlamydiaceae* and CLOs are marked (light and dark purple, respectively). Sequences of cases (purple) and controls (dark purple) of children (cTF and cC, respectively), and cases (dark blue) and controls (cyan) of adults (aTT and aC, respectively), are marked.

**Figure 3:**
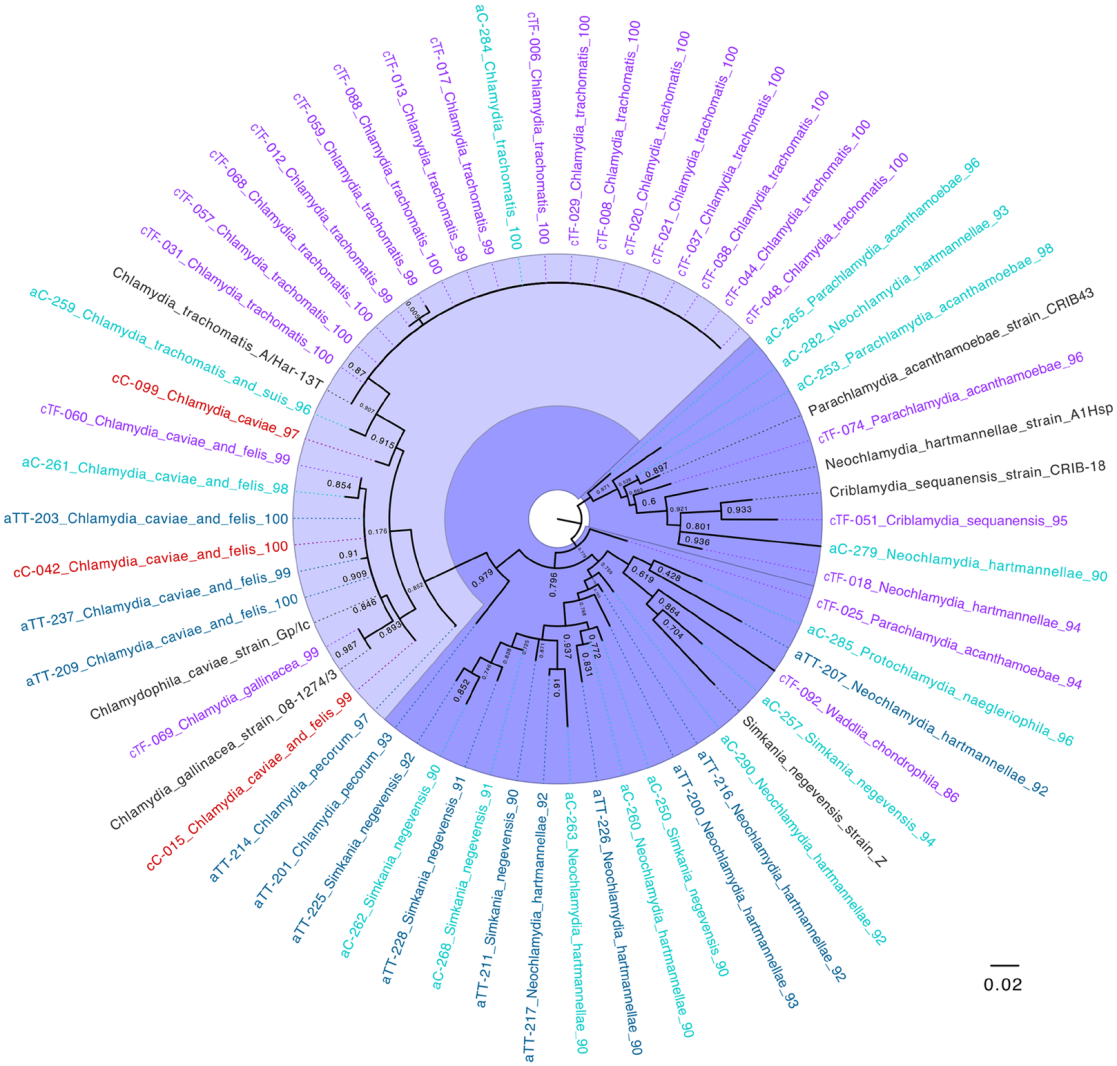
The maximum likelihood phylogenetic tree derived from the partial 16 S rRNA sequences detected in this study. Household IDs of samples are shown on left. Children cases are marked in purple (cTF), children controls (cC) in red, adults cases in dark blue (aTT), adults controls in cyan (aC), and the reference strains corresponding to species for which there was at least one best BLAST hit in black. The best BLAST hit identity (%) for each sample stands on the right side of the lables.

Among 189 swab samples in total, 68 (36%) were positive for *Chlamydiae* DNA. All the nucleic acid extraction negative controls and real-time polymerase chain reaction (PCR) negative controls remained negative when tested with *Chlamydiae* real-time PCR. Although no swab has been taken as a field control, the risk of contamination can be ruled out because of existing differences in the distribution of positive samples for *Chlamydiae* real-time PCR and various profiles of sequences corresponding to different *Chlamydiae* families among children and adults as well as cases and controls. There were no statistically significant differences in positivity for *Chlamydiae* or *C*. *trachomatis* between male and female participants (p > 0.05). The average threshold cycle (C_T_) value for *Chlamydiae* real-time PCR was 34 ± 9.4. A standard curve was prepared to evaluate the efficiency of real-time PCR and to achieve a comparative concentration (inclusion forming units (IFUs)) for the samples based on their C_T_ values (Supplementary Fig. S1). Among the 68 positive samples, 55 (81%) yielded assembled forward and reverse sequences (~200 bp) that were used for BLAST-n analysis, Naïve Bayesian classification in RDP, and phylogenetic analysis (Table 2, Figs 2 and 3) (Supplementary Tables S1 and S2). We were not able to assemble the sequences of the 13 remaining samples owing to multiple peaks in chromatogram lanes, which either might suggest the presence of more than one member of *Chlamydiae* in the ocular swab, or low amounts of DNA in the sample, which can cause PCR-generated artefactual species variation^38–41^.

**Table 2:** BLAST-n analysis and RDP classification of *Chlamydiae* 16 S rRNA sequences detected in ocular swabs of children and adults.

Household ID	% Best BLAST hit^*^ (Accession number)	% Closest BLAST match for 16S rRNA gene	Naïve Bayesian classification (RDP, 70% confidence interval)	Accession number^#^
**Children Case**				
cTF-006	100% *Chlamydia trachomatis* (CP020537.1)	100% *Chlamydia trachomatis*	*Chlamydia*	
cTF-008	100% *Chlamydia trachomatis* (CP020537.1)	100% *Chlamydia trachomatis*	*Chlamydia*	
cTF-012	99% *Chlamydia trachomatis* (CP020537.1)	99% *Chlamydia trachomatis*	*Chlamydia*	MH119791
cTF-013	99% *Chlamydia trachomatis* (CP020537.1)	99% *Chlamydia trachomatis*	*Chlamydia*	MH119792
cTF-017	99% *Chlamydia trachomatis* (CP020537.1)	99% *Chlamydia trachomatis*	*Chlamydia*	MH119793
cTF-018	95% *Neochlamydia* SP. (LC122507.1)	94% *Parachlamydiaceae*	*Neochlamydia*	MH119794
cTF-020	100% *Chlamydia trachomatis* (CP020537.1)	100% *Chlamydia trachomatis*	*Chlamydia*	
cTF-021	100% *Chlamydia trachomatis* (CP020537.1)	100% *Chlamydia trachomatis*	*Chlamydia*	
cTF-025	95% *Chlamydiales* bacterium CRIB 32 (EU363464.1)	94% *Parachlamydiaceae*	Unclassified *Parachlamydiaceae*	MH119795
cTF-029	100% *Chlamydia trachomatis* (CP020537.1)	100% *Chlamydia trachomatis*	*Chlamydia*	
cTF-031	100% *Chlamydia trachomatis* (CP020537.1)	100% *Chlamydia trachomatis*	*Chlamydia*	
cTF-037	100% *Chlamydia trachomatis* (CP020537.1)	100% *Chlamydia trachomatis*	*Chlamydia*	
cTF-038	100% *Chlamydia trachomatis* (CP020537.1)	100% *Chlamydia trachomatis*	*Chlamydia*	
cTF-044	100% *Chlamydia trachomatis* (CP020537.1)	100% *Chlamydia trachomatis*	*Chlamydia*	
cTF-048	100% *Chlamydia trachomatis* (CP020537.1)	100% *Chlamydia trachomatis*	*Chlamydia*	
cTF-051	*95% Criblamydia sequanensis* (NR_115696.1)	95% *Criblamydia*	*Parachlamydia*	MH119796
cTF-057	100% *Chlamydia trachomatis* (CP020537.1)	100% *Chlamydia trachomatis*	*Chlamydia*	
cTF-059	100% *Chlamydia trachomatis* (CP020537.1)	99% *Chlamydia trachomatis*	*Chlamydia*	
cTF-060	99% *Chlamydia caviae* (NR_074946.1)/*felis* (JN606073.1)	99% *Chlamydia caviae*/ *felis*	*Chlamydia*	MH119797
cTF-068	100% *Chlamydia trachomatis* (CP020537.1)	100% *Chlamydia trachomatis*	*Chlamydia*	
cTF-069	99% *Chlamydia gallinacea* (KX603685.1)	99% *Chlamydia gallinacea*	*Chlamydia*	MH119798
cTF-074	97% *Parachlamydia acanthamoebae* (JN051144.1)	96% *Parachlamydia*	*Parachlamydia*	MH119799
cTF-088	100% *Chlamydia trachomatis* (CP020537.1)	100% *Chlamydia trachomatis*	*Chlamydia*	
cTF-092^§^	92% *Chlamydiales* bacterium CRIB33 (EU683887.1)	86% *Chlamydiales*	Unclassified *Parachlamydiaceae*	
cTF-100	*Chlamydiales* (Failed assembly)	*Chlamydiales* (Failed assembly)	*Chlamydiales* (Failed assembly)	
**Children Control**				
cC-005	*Chlamydiales* (Failed assembly)	*Chlamydiales* (Failed assembly)	*Chlamydiales* (Failed assembly)	
cC-015	99% *Chlamydia caviae* (NR_074946.1)/*felis* (JN606073.1)	99% *Chlamydia caviae*/ *felis*	*Chlamydia*	MH119789
cC-033	*Chlamydiales* (Failed assembly)	*Chlamydiales* (Failed assembly)	*Chlamydiales* (Failed assembly)	
cC-042	100% *Chlamydia caviae* (NR_074946.1)/*felis* (AP006861.1)	100% *Chlamydia caviae*/ *felis*	*Chlamydia*	
cC-056	*Chlamydiales* (Failed assembly)	*Chlamydiales* (Failed assembly)	*Chlamydiales* (Failed assembly)	
cC-096	*Chlamydiales* (Failed assembly)	*Chlamydiales* (Failed assembly)	*Chlamydiales* (Failed assembly)	
cC-099	98% *Chlamydia* sp. 2742–308 (CP014639.1)	97% *Chlamydia caviae*	Unclassified *Chlamydiaceae*	MH119790
**Adults Case**				
aTT-200	95% *Chlamydiales* bacterium NS11 (JN606074.1)	93% *Parachlamydiaceae*	*Neochlamydia*	MH119778
aTT-201	95% *Chlamydiales* bacterium NS11 (JN606074.1)	93% *Chlamydiaceae*	Unclassified *Chlamydiales*	MH119779
aTT-203	100% *Chlamydia caviae* (NR_074946.1)/*felis* (JN606073.1)	100% *Chlamydia caviae*/ *felis*	*Chlamydia*	
aTT-207	92% *Neochlamydia hartmannellae* (NR_025037.1)	92% *Parachlamydiaceae*	*Neochlamydia*	MH119780
aTT-209	100% *Chlamydia caviae* (NR_074946.1)/*felis* (AP006861.1)	100% *Chlamydia caviae*/ *felis*	*Chlamydia*	
aTT-211	98% *Chlamydiales* bacterium CRIB 32 (EU363464.1)	90% *Simkaniaceae*	Unclassified *Chlamydiales*	MH119781
aTT-214	97% *Chlamydia* sp. (CP014639.1)/ *Chlamydia pecorum* (CP004033.1)	97% *Chlamydia pecorum*	*Chlamydophila*	MH119782
aTT-216	97% *Chlamydiales* bacterium CRIB 32 (EU363464.1)	92% *Parachlamydiaceae*	*Neochlamydia*	MH119783
aTT-217	98% *Chlamydiales* bacterium NS11 (JN606074.1)	92% *Parachlamydiaceae*	Unclassified *Parachlamydiaceae*	MH119784
aTT-219	*Chlamydiales* (Failed assembly)	*Chlamydiales* (Failed assembly)	*Chlamydiales* (Failed assembly)	
aTT-225	95% *Chlamydiales* bacterium NS11 (JN606074.1)	92% *Simkaniaceae*	Unclassified *Chlamydiales*	MH119785
aTT-226	95% *Chlamydiales* bacterium CRIB 32 (EU363464.1)/ NS11 (JN606074.1)	90% *Parachlamydiaceae*	Unclassified *Chlamydiales*	MH119786
aTT-228	96% *Chlamydiales* bacterium NS11 (JN606074.1)	91% *Simkaniaceae*	Unclassified *Chlamydiales*	MH119787
aTT-236	*Chlamydiales* (Failed assembly)	*Chlamydiales* (Failed assembly)	*Chlamydiales* (Failed assembly)	
aTT-237	99% *Chlamydia caviae* (NR_074946.1)/ *felis* (AP006861.1)	99% *Chlamydia caviae*/ *felis*	Unclassified *Chlamydiaceae*	MH119788
aTT-298	*Chlamydiales* (Failed assembly)	*Chlamydiales* (Failed assembly)	*Chlamydiales* (Failed assembly)	
**Adults Control**				
aC-250	97% *Chlamydiales* bacterium CRIB 32 (EU363464.1)	90% *Simkaniaceae*	Unclassified *Chlamydiales*	MH119764
aC-252	*Chlamydiales* (Failed assembly)	*Chlamydiales* (Failed assembly)	*Chlamydiales* (Failed assembly)	
aC-253	98% *Parachlamydia acanthamoebae* (NR_026357.1)	98% *Parachlamydia acanthamoebae*	*Parachlamydia*	MH119765
aC-257	94% *Simkania negevensis* (NR_074932.1)	94% *Simkaniaceae*	Unclassified *Chlamydiales*	MH119766
aC-258	*Chlamydiales* (Failed assembly)	*Chlamydiales* (Failed assembly)	*Chlamydiales* (Failed assembly)	
aC-259	96% *Chlamydia trachomatis* (CP019387.1)	96% *Chlamydia*	Unclassified *Chlamydiaceae*	MH119767
aC-260	97% *Chlamydiales* bacterium CRIB 32 (EU363464.1)	90% *Parachlamydiaceae*	Unclassified *Parachlamydiaceae*	MH119768
aC-261	98% *Chlamydia caviae* (NR_074946.1)/*felis* (JN606073.1)	98% *Chlamydia caviae*/ *felis*	Unclassified *Chlamydiaceae*	MH119769
aC-262	93% *Chlamydiales* bacterium CRIB 32 (EU363464.1)	90% *Simkaniaceae*	Unclassified *Chlamydiales*	MH119770
aC-263	97% *Chlamydiales* bacterium NS11 (JN606074.1)	90% *Parachlamydiaceae*	*Neochlamydia*	MH119771
aC-264	*Chlamydiales* (Failed assembly)	*Chlamydiales* (Failed assembly)	*Chlamydiales* (Failed assembly)	
aC-265	95% *Neochlamydia* sp. (LN995859.1)	93% *Parachlamydiaceae*	Unclassified *Parachlamydiaceae*	MH119772
aC-268	98% *Chlamydiales* bacterium CRIB 32 (EU363464.1)	91% *Simkaniaceae*	Unclassified *Chlamydiales*	MH119773
aC-270	*Chlamydiales* (Failed assembly)	*Chlamydiales* (Failed assembly)	*Chlamydiales* (Failed assembly)	
aC-279	90% *Neochlamydia hartmannellae* (NR_025037.1)	90% *Parachlamydiaceae*	Unclassified *Chlamydiales*	MH119774
aC-281	*Chlamydiales* (Failed assembly)	*Chlamydiales* (Failed assembly)	*Chlamydiales* (Failed assembly)	
aC-282	94% *Neochlamydia* sp. (LN995859.1)	93% *Parachlamydiaceae*	Unclassified *Parachlamydiaceae*	MH119775
aC-284	100% *Chlamydia trachomatis* (CP020537.1)	100% *Chlamydia trachomatis*	*Chlamydia*	
aC-285	96% *Protochlamydia naegleriophila* (LN879502.1)	96% *Protochlamydia*	Unclassified *Parachlamydiaceae*	MH119776
aC-290	95% *Chlamydiales* bacterium CRIB 32 (EU363464.1)	92% *Parachlamydiaceae*	Unclassified *Chlamydiales*	MH119777

^*^Uncultured bacteria were excluded from final BLAST hits by choosing the “Exclude” option in BLAST-n page for “Uncultured/environmental sample sequences”.

^#^Accession numbers for submitted sequences to the NCBI database (https://www.ncbi.nlm.nih.gov/genbank/). ^§^GenBank accession number was not provided for the cTF-092 because the BLAST query coverage was less than 90% to other Chlamydiales 16S rRNA.

BLAST-n results for sequences were categorized based on the first (Table 2) and second BLAST-hit identification (excluding uncultured bacteria) (Supplementary Table S2) and closest 16 S ribosomal RNA (16S rRNA) classification match (Table 2). Of 16 samples that were detected as *Chlamydiales* bacterium by first BLAST-hit identification, nine sequences had highest similarity to *Chlamydiales* bacterium CRIB 32 and CRIB 33, six to *Chlamydiales* bacterium NS11, and one to both *Chlamydiales* bacterium CRIB 32/NS11 (Table 2). Sequences for *Chlamydiales* bacterium CRIB 32/33 and *Chlamydiales* bacterium NS11 were previously identified in domestic water and human nasal samples, respectively^42,43^.

**Figure 4:**
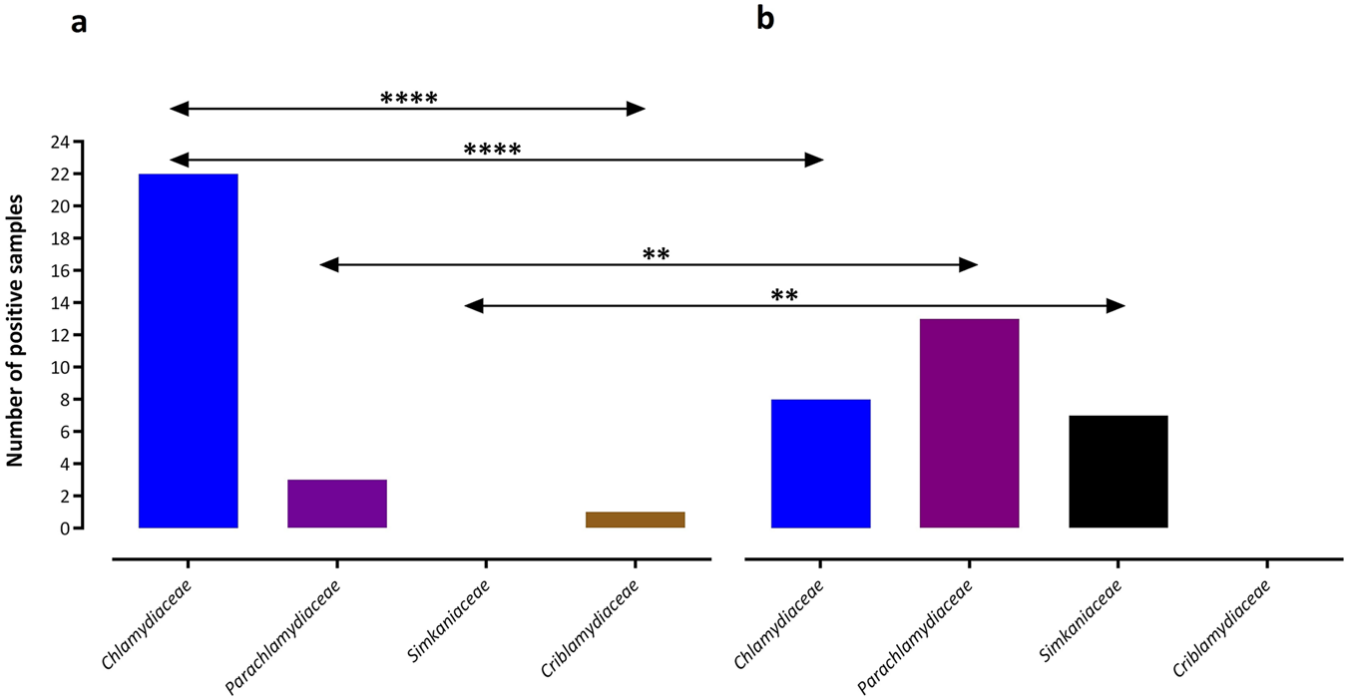
Abundance of 16 S rRNA gene sequences in the ocular samples taken from Al Qadarif-Sudan classified at the family-level in the *Chlamydiae* based on the closest BLAST hit. (**a**) Distribution of sequences obtained from children, assigned to members of chlamydial families. (**b**) Distribution of sequences obtained from adults, assigned to members of chlamydial families. The statistical significance is indicated as follows: **p < 0.01, and ****p < 0.0001.

**Figure 5:**
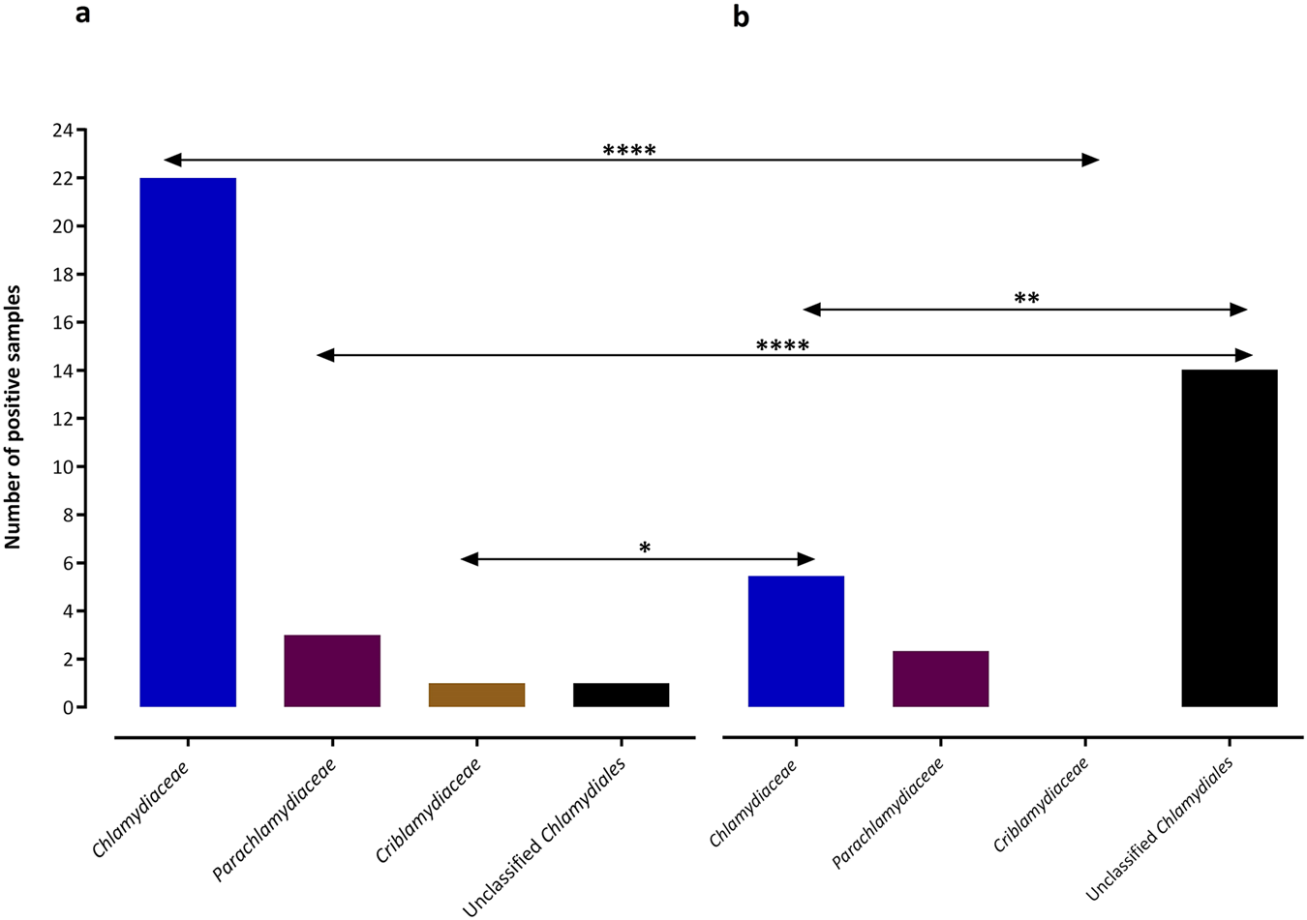
Abundance of 16 S rRNA gene sequences in the ocular samples taken from Al Qadarif-Sudan at the family-level in the *Chlamydiae* based on phylogenetic analysis. (**a**) Sequences derived from samples of children. (**b**) Sequences derived from samples of adults. The statistical significance is indicated as follows: *p < 0.05, **p < 0.01, and ****p < 0.0001.

Based on this 16S rRNA classification, four sequences corresponded to new species levels, 21 to new genus levels, and one to a new family level of the phylum *Chlamydiae*. Altogether, 54 out of 55 assembled sequences were identified as the nearest neighbor to four families including *Chlamydiaceae* (55.6%), *Parachlamydiaceae* (29.6%), *Simkaniaceae* (13%), and *Criblamydiaceae* (1.9%) (Table 2). Additionally, the retrieved 16S rRNA gene sequences were classified using the Naïve Bayesian Classifier of RDP, by which the majority of the sequences were also placed within *Chlamydiaceae* (52.7%) and *Parachlamydiaceae* (27.3%). Owing to length, the remaining sequences could only be assigned as unclassified *Chlamydiales* (20%) (Table 2). In general, the prevalence of *Chlamydiaceae* sequences was significantly higher in children than in adults (p = 0.0069) and CLO sequences were more frequent in adults than in children (p = 0.0003) (Figs 4 and 5).

### Phylogenetic analysis of sequences

Phylogenetic analyses were performed with two different datasets to obtain better insights into the taxonomic placement of the 16S rRNA sequences from this study. The sequences obtained in this study were added to a Bayesian inference tree of full-length chlamydial 16S rRNA gene sequences representing the major known chlamydial lineages using Parsimony (Fig. 2) (Supplementary Table S1 and Supplementary Fig. S2). Similar to BLAST-n results, the majority of sequences were assigned to the *Chlamydiaceae* (52.7%). The sequences affiliating with CLOs grouped together with sequences of members of the *Parachlamydiaceae* (9.1%) and *Criblamydiaceae* (1.8%). A total of 36.3% of the sequences were closely related to as yet unclassified chlamydial sequences and reflect the high diversity within the *Chlamydiae*. A maximum likelihood phylogenetic tree (Fig. 3) revealed the phylogenetic relationship of the obtained 16S rRNA gene sequences to their closest BLAST-hit including these sequences along with some reference sequences.

### Prevalence of Chlamydiae DNA in children

**Figure 6:**
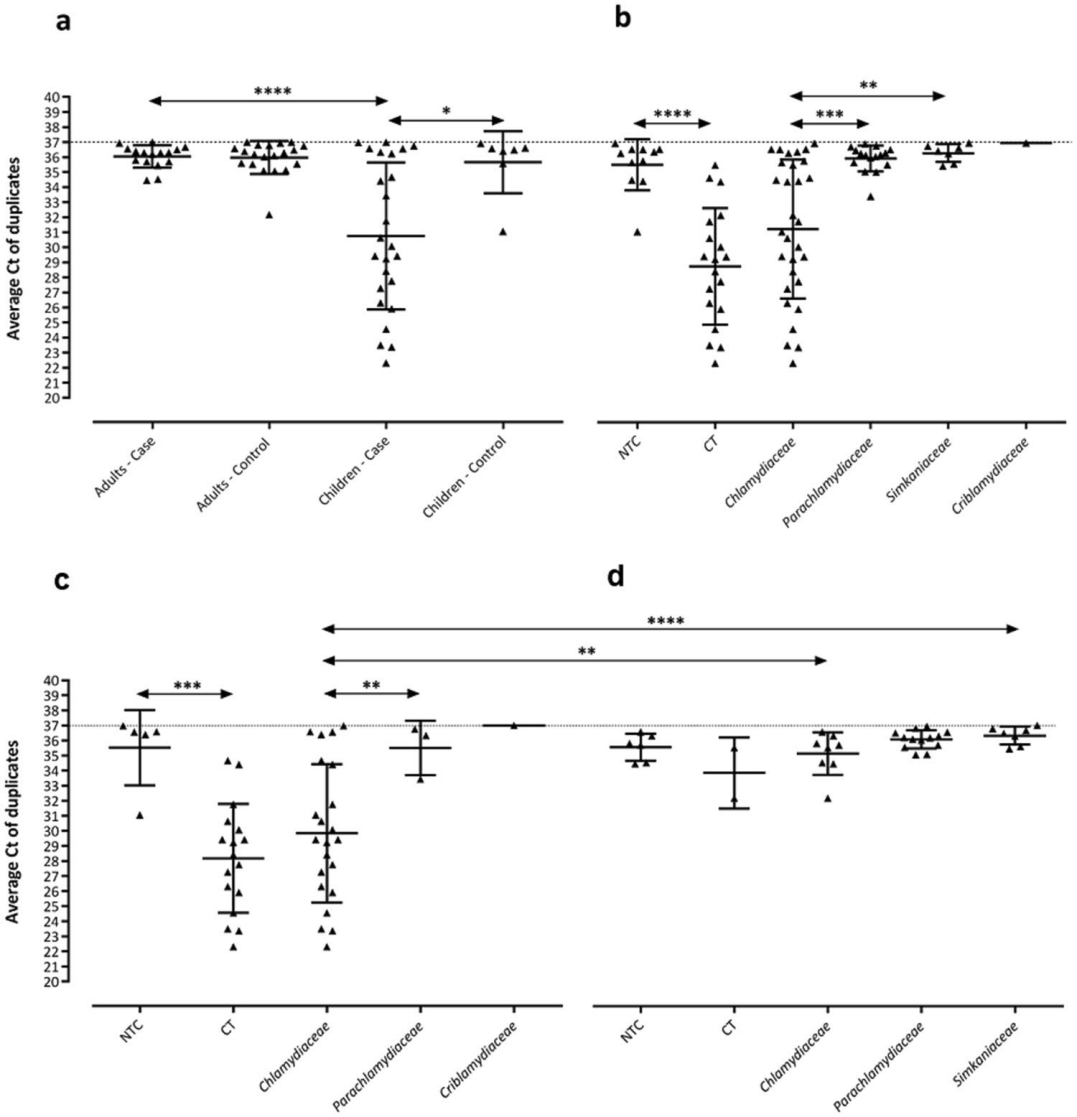
Average real-time PCR threshold cycle (C_T_) values for amplification of the *Chlamydiae* 16 S rRNA gene. (**a**) Recorded C_T_ values for positive samples among children and adults. The children case group showed significantly lower C_T_ values compared to the children control group and adults case and control groups. (**b**) Recorded C_T_ values for 16 S rRNA sequences classified as families in the phylum *Chlamydiae*, *C*. *trachomatis* (CT), and non-*trachomatis Chlamydiaceae* (NTC). C_T_ values were significantly lower for the *Chlamydiaceae* family compared to *Parachlamydiaceae*, *Simkaniaceae*, and *Criblamydiaceae* families. C_T_ values for positive samples for *C*. *trachomatis* were recorded as significantly lower than those for NTC positive samples. **(c)** Recorded C_T_ values for 16 S rRNA sequences classified as families in the phylum *Chlamydiae*, CT, and NTC among children. **(d)** Recorded C_T _values for 16 S rRNA sequences classified as families in the phylum *Chlamydiae*, CT, and NTC among adults. The statistical significance is indicated as follows: *p < 0.05, **p < 0.01, ***p < 0.001, and ****p < 0.0001.

Of 96 samples from children (54 in the trachomatous inflammation-follicular (cTF) group and 42 in the control (cC) group), 32 (33.3%) yielded positive results for *Chlamydiae* (25 in the cTF group and 7 in the cC group). The prevalence of *Chlamydiae* DNA in the cTF group (45.3%) was significantly higher than that in the cC group (16.7%) (p = 0.0032). The average C_T_ values in the cTF and cC groups tested using *Chlamydiae* real-time PCR were 30.8 ± 9.2 and 35.7 ± 0.7, respectively (p = 0.0154) (Fig. 6a).

Among the 32 positive samples, 27 sequences were assembled. Sequences from 26 samples corresponded to various families: 22 (84.6%) were positive for *Chlamydiaceae*, three (11.5%) for *Parachlamydiaceae*, and one for (3.8%) *Criblamydiaceae* (Table 2 and Fig. 4a). In addition, one sample (cTF092) exhibited 87% similarity to the order *Chlamydiales* and appeared to be phylogenetically close to unclassified *Chlamydiales* (Table 2 and Fig. 2).

In the cTF group, among the 19 sequences classified as *Chlamydiaceae*, 17 (89.5%) showed 99–100% homology to *C*. *trachomatis* (p < 0.0001). These results were confirmed using *omcB* real-time PCR. In general, *C*. *trachomatis* sequences had a frequency of 31.5% (17/54) among the cTF group. The remaining two positive samples showed sequence homology with *Chlamydia caviae/Chlamydia felis* and *Chlamydia gallinacea* and remained negative when tested with *omcB* real-time PCR. Among three assembled sequences in the cC group, two revealed highest similarity to both *C*. *caviae*/*C*. *felis* and one to only *C*. *caviae* (Table 2). None of the samples in the cC group exhibited similarities to *C*. *trachomatis* or had positive testing results using *omcB* real-time PCR. Moreover, among the three sequences classified as *Parachlamydiaceae* in the cTF group, two sequences showed highest similarity to *Parachlamydia* and one to *Neochlamydia*. In addition, one sample (cTF051) was classified as *Criblamydiaceae* (Table 2). This is also reflected in the phylogenetic trees (Figs 2 and 3).

**Figure 7:**
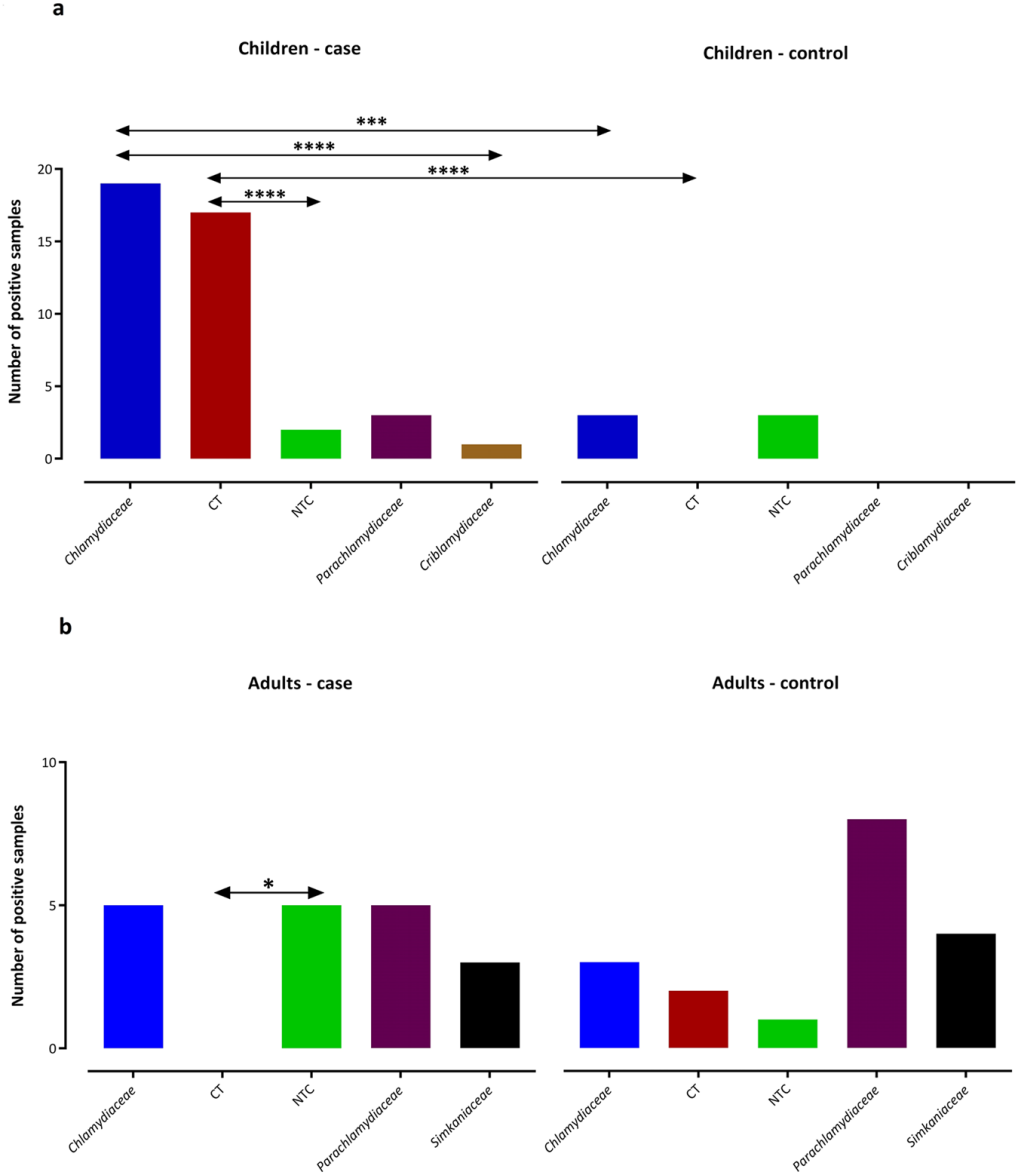
Distribution of 16 S rRNA gene sequences assigned to families in the phylum *Chlamydiae*, *C*. *trachomatis* (CT), and non-*trachomatis Chlamydiaceae* (NTC) among (**a**) children and (**b**) adults. Prevalence of classified sequences as CT in the children case group was significantly higher than that in the children control group. The statistical significance is indicated as follows: *p < 0.05, ***p < 0.001, and ****p < 0.0001.

Altogether, *C*. *trachomatis* comprised the majority of 16S rRNA sequences compared to non-*trachomatis Chlamydiaceae* (NTC) and all CLOs among children (*C*. *trachomatis* vs. NTC and CLOs, p = 0.0005 and p = 0.0001, respectively) (Fig. 7a). The frequency of *Chlamydiaceae* sequences was significantly higher than that of all classified or unclassified CLOs among the children samples (p < 0.0001) (Figs 4a and 5a). No significant differences were found between cTF and cC groups with respect to the affiliation to different CLO families (p > 0.05) (Fig. 7a).

### Prevalence of Chlamydiae DNA in adults

Overall, 36 out of 93 (38.7%) swabs taken from adults tested positive for *Chlamydiae* DNA. Prevalence of *Chlamydiae* in the adult control (aC) group (20/42) was higher than that in adults with trachomatous trichiasis (aTT) (16/51); however, this difference was not significant (p > 0.05). The average C_T_ values in the aTT and aC groups tested using *Chlamydiae* real-time PCR were 36.0 ± 0.45 and 36 ± 0.8, respectively (p > 0.05) (Fig. 6a). The majority of sequences from adults were identified by BLAST search as being similar to the *Parachlamydiaceae* family (13 sequences, 46.4%), eight (28.6%) similar to the *Chlamydiaceae* and seven (25%) to the *Simkaniaceae* family (Table 2 and Fig. 4b). Phylogenetic analysis of the sequences from adults, including many lineages of yet uncultured chlamydial species, revealed three main groups including unclassified *Chlamydiales* (64.3%), *Chlamydiaceae* (25%), and *Parachlamydiaceae* (10.7%) (Fig. 5b).

The distribution of *Chlamydiaceae* sequences within the aTT group consisted of one sequence that showed highest similarity to genus *Chlamydia*, one to *C*. *pecorum*, and three that showed equivalent identification as either *C*. *caviae* or *C*. *felis*. With regard to the three controls positive for the *Chlamydiaceae* family, two sequences revealed highest similarity to *C*. *trachomatis* and also tested positive in *omcB* real-time PCR. The remaining sample exhibited equivalent similarity to *C*. *caviae/C*. *felis* (Table 2).

In general, the number of sequences classified as CLOs within the adult group was significantly higher than that classified as *Chlamydiaceae* (p = 0.0137) (Figs 4b and 5b). Nevertheless, no significant differences were found between aTT and aC groups with respect to the affiliation to different *Chlamydiae* families (p > 0.05) (Fig. 7b).

## Discussion

This study provided evidence for the presence of *Chlamydiaceae* and CLO DNA in human ocular samples of patients with trachoma and healthy controls in Sudan. Overall, 36% of all samples tested positive for *Chlamydiae* DNA. Whereas the prevalence of *C*. *trachomatis* was significantly higher in children than in adults, a significantly higher distribution of CLO DNA was detected in adult samples compared to those from children. However, based on differences in the occurrence of identified CLO sequences among the patients with trachoma and healthy controls in children and adults, a pathogenic role for these bacteria in trachoma is not evident.

To our knowledge, this study is the first to examine human ocular samples for the presence of all members of the *Chlamydiae*. Whereas 22.9% of children’ and 8.6% of adults’ samples corresponded to *Chlamydiaceae* sequences, amplicons of 21.5% of adults’ samples and 4.2% of children samples corresponded to sequences originating from different CLOs. These results are comparable with a prior study on cats with and without (controls) ocular diseases^16^. Specifically, *C*. *felis*, the known pathogen for feline chlamydial conjunctivitis^44^, was found among the majority of diseased eyes in animals under the age of five. The distribution of positive samples for non-*C*. *felis Chlamydiales* increased from 30% for cats under the age of one to 54% for cats older than 10 years in animals with and without ocular diseases^16^. Furthermore, in two separate studies, in nasopharyngeal samples from healthy children and adults with and without pneumonia, 11.4% of children samples and 36% of samples from adults tested positive for members of various families in the phylum *Chlamydiae*^43,45^. It is not clear why the distribution of CLOs in adults was more than five times higher than that in children. Although this difference might constitute a bias resulting from the small sample size in each group, this does not appear likely.

In the present study, we found a higher diversity of CLOs in adults than in children. Some of the analyzed sequences belong to members of the *Parachlamydiaceae*, which have previously been detected in other mammalian tissues^46^. *Parachlamydia* spp. have been found directly and indirectly in human corneal samples and disposable contact lenses, respectively^19,47^. To our knowledge, this is the first report of *Criblamydiaceae* in human eyes. The majority of sequences obtained, however, were affiliated with various as yet uncharacterized chlamydial lineages. This is unexpected, because these lineages are generally to date represented only by environmental clone sequences, with members of such lineages not having been considered to be also associated with humans. Although the sequences belonging to these divergent CLOs have mainly been detected in healthy participants and may also originate from other sources, it is still noteworthy to find such a diversity of CLOs sequences in human eyes. Considering that similar profiles of CLOs were obtained among the aTT and aC groups, we cannot suggest any association of these bacteria with trachoma in adults. In children, the distribution of CLOs did not significantly differ between the cTF and cC groups.

Several studies have reported the pathogenic impacts of *Parachlamydia* spp. in respiratory disorders^6,20,21,26^ and *S*. *negevenesis* in patients with bronchiolitis, although *S*. *negevenesis* pathogenic potential remains controversial^6,20,21,48–50^. Notably, the possible role of some CLOs in respiratory tract infections was mainly suspected in pediatric populations; similarly, in the present study, the only limited evidence suggesting a role of *Parachlamydiaceae* and *Criblamydiaceae* in trachoma pathogenesis was documented in children.

In our study, no strong evidence of the pathogenic impact of CLOs on adult human eyes was obtained, which supports previous results by Vidgen *et al*. suggesting CLOs as commensals of the ocular microbiota in koala^51^. Estimated loads of CLOs in this study are comparable with the reported load of the bona fide microbiome: 100 to 1000 colony-forming unit (CFU) per mL in human tear fluid^52^. These results are in line with several studies reporting a non-significant distribution of *Parachlamydiaceae* and other CLOs among diseased and healthy eyes of sheep, pigs, and cats^16–18,53^. Moreover, CLOs have been detected in different samples such as skin, nose, and cervicovaginal samples of healthy individuals^22,38,43,45^.

The high prevalence of *Chlamydiae* DNA in the present study is comparable with findings of human skin and nasal samples, which support the idea that ocular microbiota colonizes in parallel with microbiota of the upper respiratory system and skin^22,43^. This high prevalence compared to results from human respiratory or cervicovaginal samples can be explained through the frequent contact of the ocular surface directly or indirectly with a source of bacteria in the environment, infected animals, and humans. In particular, the majority of unclassified chlamydial sequences in this study demonstrated highest homology to previously isolated sequences from water. Additionally, members of *Parachlamydiaceae* are known to naturally infect amoeba, which they utilize as a replicative niche^23,42,54–56^. Moreover, *Simkania* and *Criblamydia* have been detected repeatedly in domestic waters^14,42,54,55,57,58^, which highlights the importance of water as a rich source of CLOs^5^ and possibly a main source of ocular infection with *Chlamydiae*. These results also emphasize the validity of environmental improvement as an essential factor of the SAFE strategy (**S**urgery for trichiasis, **A**ntibiotics to clear ocular infection, promotion of **F**acial cleanliness, and **E**nvironmental improvement).

The profile of the *Chlamydiaceae* community present in ocular mucosa differed significantly between adults and children. Whereas *C*. *trachomatis* was only detected in two (2.1%) adult samples, 17 samples (31.5%) of children with TF were found to be positive for *C*. *trachomatis*. These results are consistent with previous data suggesting a central role for *C*. *trachomatis* in active trachoma among children^1,59,60^. Notably, the only two samples from adults that yielded positive results for *C*. *trachomatis* infection in the present study were in the aC group. The absence of infection with *C*. *trachomatis* in adults with trachoma can be explained through: [1] the effects of non-*C*. *trachomatis* bacteria and mechanical irritation on the eyes^30–32,34,61,62^; [2] the development of acquired immunity to *C*. *trachomatis* and its impact on the duration of ocular infection, which decreases significantly with age^28,34,59,61,63^; and [3] the kinetics of the disease, which can be explained by the recovery phase after repeated infections with *C*. *trachomatis*^60^. During this time, patients can be diagnosed positive for trachoma although *Chlamydia* is no longer present in the eyes^60^.

Classification of the *Chlamydiaceae* positive sequences into *C*. *trachomatis* and NTC provided an opportunity to investigate the prevalence of NTC bacteria. Overall, we found no association between NTC species and trachoma in this population. Although NTC species in the aTT group (9.8%) compared to the aC group (2.4%) were more frequent, this difference was not statistically significant. This is in contrast to previous studies, which found a high prevalence of NTC species in trachoma samples from Nepal^28,29^. Differences in NTC prevalence between present data and previous studies by Dean *et al*.^28,29^ could be due to the small sample size in all of these studies and regional variations.

Unfortunately, BLAST results for the majority (8/11, 72.7%) of sequences positive for NTC species could not differentiate between *C*. *caviae* and *C*. *felis* as the same identification score was obtained for both species. Moreover, the non-significant distribution of sequences positive for *C*. *caviae*/*C*. *felis* between cases compared to controls was unable to provide a clear indication of their role in this study. *C*. *caviae* and *C*. *felis* cause keratoconjunctivitis in guinea pigs and cats, respectively^16,19,28,64,65^. Previous studies have also reported the presence of *C*. *caviae* and *C*. *felis* in human eyes^19,66,67^. In our study, one sequence obtained in the aTT group and one in the cTF group demonstrated highest homology to *C*. *pecorum* and *C*. *gallinacea*, respectively. The presence of *C*. *pecorum* in an ocular sample from a patient with trachoma has also been previously reported^29^. To the best of our knowledge, this is the first molecular evidence of the presence of *C*. *gallinacea* DNA in humans, a species recently added to the *Chlamydiaceae* that was reported for the first time in cattle by Li *et al*.^68^ and subsequently described as a causative agent of avian chlamydiosis with zoonotic potential^69–71^.

C_T_ values revealed important information about the bacterial load in each sample. The average C_T_ value for children infected with *C*. *trachomatis* corresponds to 1.000 to 10.000 IFU per swab (Fig. 6c), whereas those for NTC and CLO positive samples correspond to 10 to 100 IFU per swab (Fig. 6b–d). These data support prior findings that active infection (higher loads) of *C*. *trachomatis* is associated with active trachoma^61,72–74^. Furthermore, consistent with previous data, we found higher loads of bacteria in samples from children^59,72,73^, suggesting this age group as the major source of *C*. *trachomatis* infection^59^. Higher C_T_ values, which are corresponding to lower loads of bacteria in positive samples for CLOs might be due to the persistent form of infection in these samples as it has been described earlier^74,75^. Nevertheless, high C_T_ values may also be the consequence of an early infection, the presence of residual DNA from a previous infection, or inoculation of the eye with dead microorganisms^28,61^.

Our study also provides evidence that the presence of CLOs and *C*. *trachomatis* in human eyes was influenced by age. Previously, 16S rRNA gene sequencing revealed a highly diverse bacterial community in human eyes^76–78^. Moreover, age-dependent changes in the ocular microbiota have been reported in prior studies^79,80^. It has been shown that the ocular microbiome of individuals with healthy conjunctiva has a significantly different bacterial abundance and diversity in children aged ≤10 compared to older participants^80^. In addition, recent studies have uncovered strong evidence regarding the impacts of ocular commensal microbiota on regulating local immune responses and developing defense mechanisms against colonization of pathogens in the eyes^52,79,81,82^. The absence of CLOs and *C*. *trachomatis* in children and adults can thus be partially explained owing to age-dependent changes in the microbiota of eyes and its impacts on the development of different local immune responses. This may in turn influence the presence of *Chlamydiae* and multiplicity of infection. For example, a study on the cervical microbiota of women infected with *C*. *trachomatis* and healthy controls have shown a different diversity of microbiota in those with asymptomatic infection of *C*. *trachomatis*^83^. Additionally, the abundance of bacterial taxa was different in the cervical microbiota of women infected with *C*. *trachomatis* compared to that in healthy controls^83^. Vidgen *et al*. have also demonstrated a correlation between urogenital microbiota of koalas and infection with *C*. *pecorum*^51^. Furthermore, previous results have shown that co-infection of *C*. *trachomatis* serovar E and another intracellular pathogen, *Toxoplasma gondii*, can lead to a stress-induced persistent growth of *C*. *trachomatis* in the host cells owing to better ability of *T*. *gondii* in scavenging nutrients inside the cells^84^. The suggestion of CLOs as comprising part of the commensal microbiota of the eye thus raises the question of whether the presence of CLOs can influence successful infection of *C*. *trachomatis* in human eyes.

This study has several limitations. As the sample size was relatively small, the use of a larger sample size within this study might have minimized the bias in biodiversity of detected CLOs in each group and provided a better understanding of the possible associations of CLOs and NTC species with eye health or disease. In addition, the length of the amplicons used for sequencing was not sufficient to resolve the species (i.e. *C*. *felis/C*. *caviae*) or genus level in every case, due to the sequence identity or low representation of many chlamydial lineages in public databases, respectively.

In summary, *C*. *trachomatis* is associated with active trachoma in Sudanese children. We presented evidence demonstrating an age-dependent distribution of divergent groups of CLOs in human eyes. However, the biology of the CLOs identified in this study remains unknown. Our findings do not support a significant association between the presence of CLOs and NTC species and active trachoma in children or TT in adults. Comparative studies with a larger sample size on the prevalence of CLOs in human eyes from different geographic regions may provide insight into the possible role of these microorganisms in pathogenicity and/or protection. Further studies are needed to understand the impacts of ocular microbiota and local immune responses on the presence of *C*. *trachomatis* and CLOs in the eyes.

## Material and Methods

### Ethical approval

The study was conducted in accordance with the Declaration of Helsinki. The National Ethics Authorities in Sudan (Sudanese National Research Ethics Review Committee (NRERC) (No. 174-8-12)) and the ethical committee of the Medical University of Vienna approved the study and all the included procedures. Written informed consent was obtained from all adult individuals at the time of sample collection. For those participants aged <18 years that wished to take part in the study, consent was obtained from a parent/guardian. All samples were anonymized.

### Trachoma grading

Clinical phenotypes were assessed in the field by an experienced ophthalmologist trained in trachoma grading using the World Health Organization simplified grading system^85^. Individuals presenting with follicular trachoma were classified as TF, individuals with pronounced papillary hypertrophy and inflammatory thickening of tarsal conjunctiva were classified as “Trachomatous Inflammation − Intense (TI)” and individuals with tarsal conjunctival scarring as “Trachomatous scarring (TS)”. Individuals with inturned eyelashes were graded as TT. Individuals with no clinical signs of follicles, papillary hypertrophy, or conjunctival scarring were classed as healthy controls. Cases and controls were matched by age and gender.

### Study population and sampling

Participants for this study were recruited in trachoma endemic areas in Sudan^86^. For adults with TT, sample collection took place in a Field Surgery Clinic in the Al Qadarif region (Fig. 1). A total of 51 aTT scheduled for trichiasis surgery and 42 matched aC without history and present signs of trachoma were enrolled. In addition, 54 cTF and 42 cC subjects aged 1 to 9 years were recruited at two Quran Schools in the Al Qadarif region. Conjunctival samples were taken from the upper tarsal conjunctiva from both eyes using polyester flocked swabs (UTM-RT collection kits, Copan USA, Murrieta, CA, USA) using standard methodology^87,88^. The sampling ophthalmologist wore gloves that were changed after each individual. Swabs were stored in universal transport medium and frozen immediately in liquid nitrogen cryogenic shipping containers. In Austria, samples were subsequently stored at −80 °C.

### DNA preparation

Genomic DNA was extracted from the conjunctival swab samples taken from left eyes. As a nucleic acid extraction negative control, one empty swab was included during each DNA extraction run. Each swab was vigorously vortexed for 5 min inside the universal transport medium, then the entire transport medium was transferred to a new tube. Cells in the swab transport medium were pelleted by centrifugation at 17,000 × *g* for 30 min. DNA was extracted from the resuspended pellet in ATL buffer of the QIAamp DNA Investigator Kit (QIAGEN GmbH, Hilden, Germany), according to manufacturer instruction, and DNA was stored at −20 °C until further use.

### Broad-range Chlamydiae real-time PCR and sequencing

A *Chlamydiae*-specific real-time PCR was performed on a final reaction volume of 20 µL targeting a fragment of approximately 207 to 215 bp of the 16S rRNA gene as described by Lienard *et al*.^45^. Cycling conditions were 5 min at 95 °C, followed by 50 repetitions of 3-step cycles of 15 s at 95 °C, 20 s at 67 °C, and 15 s at 72 °C, all carried out in a PikoReal real-time PCR System (Thermo Fisher Scientific). All samples were tested in duplicate. After optimization of the PCR, a C_T_ value of ≤37 has been suggested as a reliable cutoff to provide reproducible results and samples with this C_T_ value were considered as positive samples. In each run, 2 wells were dedicated to negative controls (distilled water) and 2 wells were used as nucleic acid extraction negative controls. A standard curve was generated for each real-time PCR run using decimal serial dilutions of extracted genomic DNA from 1 × 10^6^ to 1 × 10^1^ IFUs of *C*. *trachomatis* (Supplementary Fig. S1).

Purification of amplicons for positive samples was performed using the QIAquick PCR Purification Kit (QIAGEN GmbH) according to the manufacturer’s protocol. Sanger sequencing of purified PCR products was performed by Eurofins Genomics AT (Vienna, Austria). Inner primers (panFseq and panRseq) resulted in an approximately 200 bp amplicon as explained previously by Lienard *et al*.^45^.

### Specific TaqMan real-time PCR for the omcB gene of C. trachomatis

To assess *C*. *trachomatis* prevalence in particular, a specific real-time PCR was carried out on all samples to target the *omcB* gene of *C*. *trachomatis*. The reaction was performed in a final volume of 20 µL using iTaq Supermix (BioRad, Reinach, Switzerland), 0.3 µM concentration of each primer, 0.1 µM concentration of probe, and 5 µL sample DNA^89^. The program was set at 95 °C for 5 min, followed by 45 cycles of 15 s at 95 °C and 30 s at 60 °C, all carried out in a PikoReal real-time PCR System. All samples were tested in duplicate. Samples with a C_T_ value of ≤37 were considered as positive. Distilled water (a negative control) and *C*. *trachomatis* DNA (a positive control) were included in each experiment.

### Data analysis

The obtained partial forward and reverse sequences of 16S rRNA gene were trimmed, aligned and assembled in the Geneious R10.2.2 software package. Of the resulting 55 sequences, 50 (90.9%) were of high quality with average phred quality scores above 30 and 5 (9.1%) of the sequences had average phred quality scores above 23 (cTF-025, aC-259, aC-261, aC-268 and aC-285). All consensus sequences were checked for the presence of chimera with DECIPHER^90^ and compared with sequences available in the GenBank database of the National Center for Biotechnology Information via the BLAST server (https://www.ncbi.nlm.nih.gov/blast/). 16S rRNA gene identification cut-offs of 97, 95, and 90% were applied for the achieved sequences to approximately classify members of the phylum *Chlamydiae* to the species, genus, and family levels, respectively^91^. In addition, the 16S rRNA sequences were classified with the Naïve Bayesian classifier of RDP using a 70% confidence interval^92^. Obtained nucleotide sequences have been submitted to the NCBI database (https://www.ncbi.nlm.nih.gov/genbank/) under accession numbers (MH119764-99).

Based on the results from sequencing of 16S rRNA gene sequences and *C*. *trachomatis omcB* real-time PCR, samples assigned to the *Chlamydiaceae* were divided into two groups: *C*. *trachomatis* and NTC.

### Phylogenetic analysis

Phylogenetic analyses were carried out with two datasets. First, a dataset contained full-length 16S rRNA gene sequences of *Chlamydiae*, which were downloaded from GenBank and aligned to the SILVA SSU Ref database containing preconfigured high-quality full-length sequences in ARB^93,94^. Bayesian inference analysis of full-length 16S rRNA gene sequences was carried out with MrBayes 3.2.6 using standard settings via the CIPRES Science Gateway^95,96^. The partial 16S rRNA gene sequences obtained in this study were added subsequently to the Bayesian tree using the Quick-Add Parsimony option in ARB^93^. The phylogenetic tree was visualized with iTOL and FigTree^97,98^ (Fig. 2).

Second, all obtained consensus sequences as well as the 16S rRNA gene sequences of *C*. *trachomatis* A/Har-13T (A_E17344.1), *C*. *caviae* strain: Gp/Ic (=ATCC VR813) (D85708.1), *N*. *hartmannellae* strain A1Hsp (NR_025037.1), *Criblamydia sequanensis* strain CRIB-18 (NR_115696.1), *S*. *negevensis* strain Z (NR_029194.1), and *P*. *acanthamoebae* strain CRIB43 (FJ532291.1) were used. Sequences were aligned using MAFFT v7.309^99^. Jalview v2.10.1 was used to trim the alignment and to look for reverse complemented sequences^100^. Maximum likelihood-based phylogenetic analyses were performed using FastTree 2.1 software (with parameters “-nt -gtr -spr 4 -mlacc 2 -slownni”)^101^. The phylogenetic representation was rerooted at the midpoint using Archaeopterix v0.9920^102^ and displayed with FigTree v1.4.2^98^ (Fig. 3).

### Statistics

Variables were compared by t test. A p-value < 0.05 was taken as statistically significant. Data analysis was performed using GraphPad Prism 6.0 (GraphPad Inc., La Jolla, CA, USA) software.

## Electronic supplementary material

Supplement

Dataset 1
