# Supplementary material for: Detection of Chlamydiaceae and Chlamydia-like organisms on the ocular surface of children and adults from a trachoma-endemic region
Source: Sci Rep. 2018 May 9;8:7432. doi: 10.1038/s41598-018-23887-1 (PMC5943520; doi:10.1038/s41598-018-23887-1)
Supplement: Supplementary file 1 — Supplement [file 41598_2018_23887_MOESM1_ESM.pdf]

Detection of *Chlamydiaceae* and *Chlamydia*-like organisms on the ocular surface of children and adults from a trachoma-endemic region

Ehsan Ghasemian[1], Aleksandra Inic-Kanada[1], Astrid Collingro[2] , Florian Tagini[3], Elisabeth Stein[1], Hadeel Alchalabi[1], Nadine Schuerer[1], Darja Keše[4], Balgesa Elkheir Babiker[5], Nicole Borel[6], Gilbert Greub[3,7], Talin Barisani-Asenbauer[1]*

[1] OCUVAC – Center of Ocular Inflammation and Infection, Laura Bassi Centres of Expertise, Center for Pathophysiology, Infectiology and Immunology, Medical University of Vienna, Vienna, Austria

[2] Department of Microbiology and Ecosystem Science, Division of Microbial Ecology, University of Vienna, Vienna, Austria

[3] Institute of Microbiology, University of Lausanne and University Hospital, Lausanne, Switzerland

[4] Institute of Microbiology and Immunology, Faculty of Medicine, University of Ljubljana, Ljubljana, Slovenia

[5] Federal Ministry of Health, Khartoum, Sudan

[6] Institute of Veterinary Pathology, Department of Pathobiology, Vetsuisse Faculty, University of Zurich, Zurich, CH-8057, Switzerland

[7] Infectious Diseases Service, University Hospital of Lausanne, Lausanne, Switzerland

*Corresponding author: Talin Barisani-Asenbauer (talin.barisani@meduniwien.ac.at)

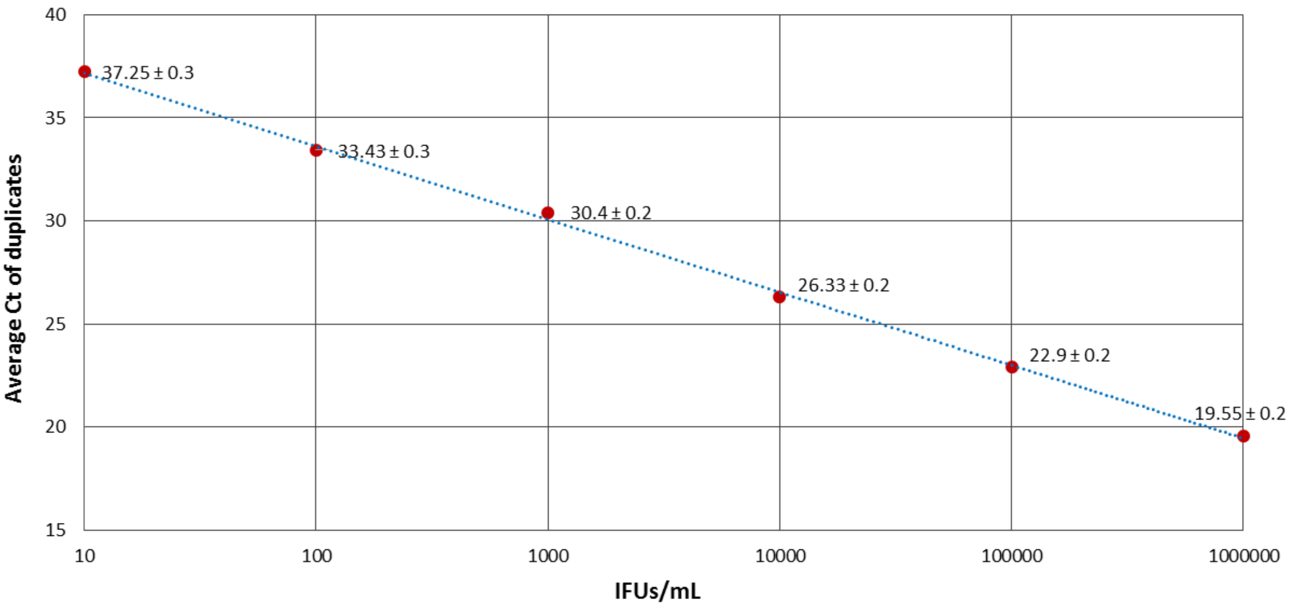

Figure S1. Sensitivity and efficiency of broad-range *Chlamydiae* real-time PCR. A series of 10-fold dilutions of defined numbers of inclusion forming units (IFUs) of *C. trachomatis* (from 1×10^6 to 1×10^1) were prepared for purification of genomic DNA. The prepared dilutions were subjected to each broad-range *Chlamydiae* real-time PCR run in duplicate. Every red dot on the graph indicates the average C_T value for the corresponding dilution factor during the study. The r^2 linearity value from the linear regression is 0.9991. (y = −3.5474×(x) + 40,726; efficiency = 91.38%).

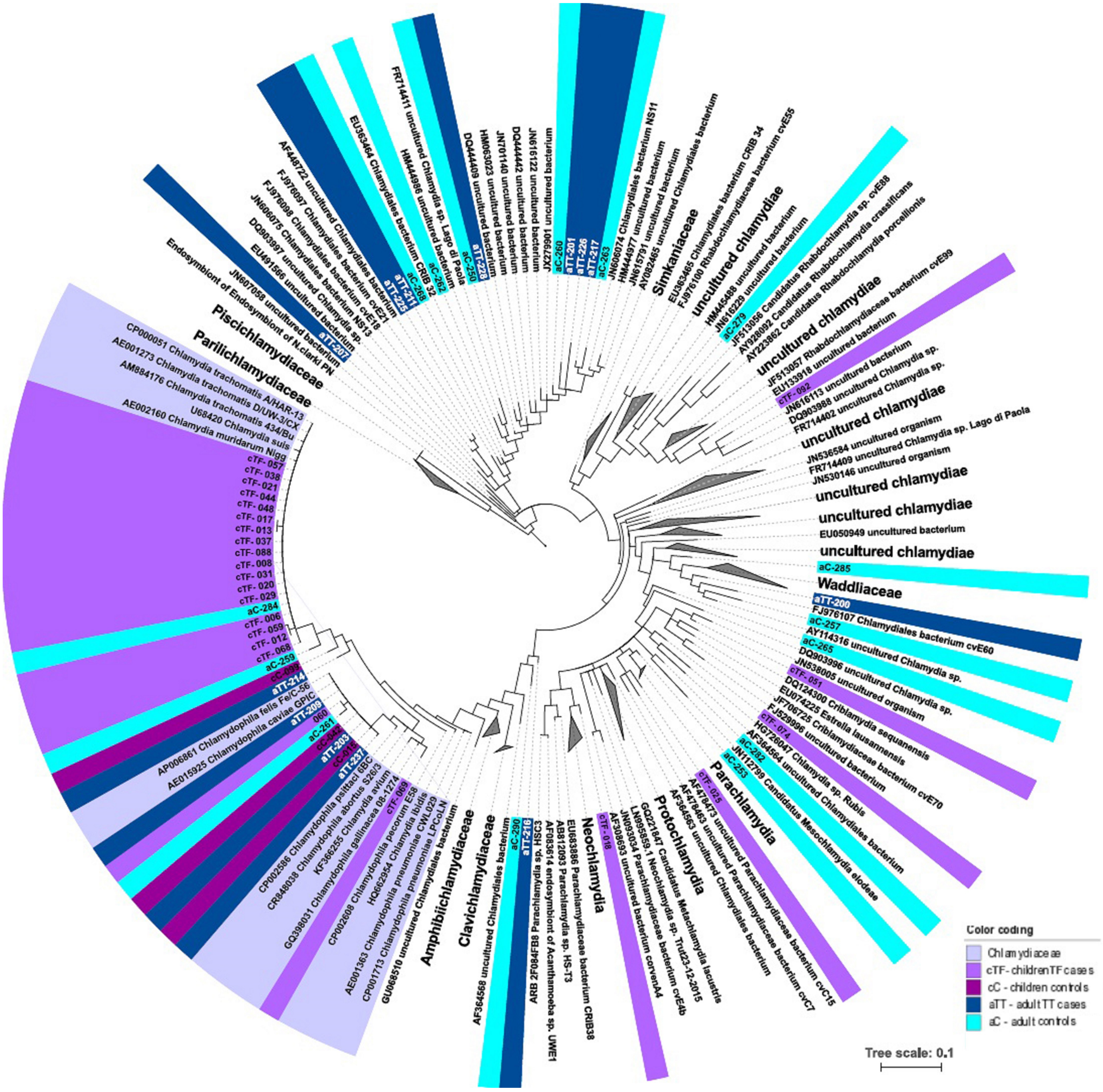

Figure S2. Phylogenetic affiliation of 16S rRNA gene sequences detected in this study to all used sequences. A Bayesian tree built from full-length chlamydial sequences is shown, to which the partial sequences generated in this study were added with Parsimony.

Table S1. Amplified 16S rRNA sequences and their taxonomic affiliation and closest related sequences in the phylogenetic tree.

Household ID	Taxonomy in phylogenetic tree	Closest related sequence in tree	% Similarity	Other sequences with same similarity
**Children Case**				
cTF-006	*Chlamydia*	CP000051, *Chlamydia trachomatis* A/HAR-13	100	AE001273, *Chlamydia trachomatis* D/UW-3/CX
cTF-008	*Chlamydia*	CP000051, *Chlamydia trachomatis* A/HAR-13	100	AE001273, *Chlamydia trachomatis* D/UW-3/CX
cTF-012	*Chlamydia*	CP000051, *Chlamydia trachomatis* A/HAR-13	99.5	AE001273, *Chlamydia trachomatis* D/UW-3/CX
cTF-013	*Chlamydia*	CP000051, *Chlamydia trachomatis* A/HAR-13	99.5	AE001273, *Chlamydia trachomatis* D/UW-3/CX
cTF-017	*Chlamydia*	CP000051, *Chlamydia trachomatis* A/HAR-13	99.5	AE001273, *Chlamydia trachomatis* D/UW-3/CX
cTF-018	*Neochlamydia*	AF098330, Endosymbiont of Acanthamoeba sp. TUME1	96	
cTF-020	*Chlamydia*	CP000051, *Chlamydia trachomatis* A/HAR-13	100	AE001273, *Chlamydia trachomatis* D/UW-3/CX
cTF-021	*Chlamydia*	CP000051, *Chlamydia trachomatis* A/HAR-13	100	AE001273, *Chlamydia trachomatis* D/UW-3/CX
cTF-025	*Parachlamydia*	AF478463, Uncultured *Parachlamydiaceae* bacterium cvC7	95.1	
cTF-029	*Chlamydia*	CP000051, *Chlamydia trachomatis* A/HAR-13	100	AE001273, *Chlamydia trachomatis* D/UW-3/CX
cTF-031	*Chlamydia*	CP000051, *Chlamydia trachomatis* A/HAR-13	100	AE001273, *Chlamydia trachomatis* D/UW-3/CX
cTF-037	*Chlamydia*	CP000051, *Chlamydia trachomatis* A/HAR-13	100	AE001273, *Chlamydia trachomatis* D/UW-3/CX
cTF-038	*Chlamydia*	CP000051, *Chlamydia trachomatis* A/HAR-13	100	AE001273, *Chlamydia trachomatis* D/UW-3/CX
cTF-044	*Chlamydia*	CP000051, *Chlamydia trachomatis* A/HAR-13	100	AE001273, *Chlamydia trachomatis* D/UW-3/CX
cTF-048	*Chlamydia*	CP000051, *Chlamydia trachomatis* A/HAR-13	100	AE001273, *Chlamydia trachomatis* D/UW-3/CX
cTF-051	*Criblamydiaceae*	JN538005, Uncultured organism	96.1	
cTF-057	*Chlamydia*	CP000051, *Chlamydia trachomatis* A/HAR-13	100	AE001273, *Chlamydia trachomatis* D/UW-3/CX
cTF-059	*Chlamydia*	CP000051, *Chlamydia trachomatis* A/HAR-13	98.9	AE001273, *Chlamydia trachomatis* D/UW-3/CX
cTF-060	*Chlamydia*	AP006861, *Chlamydia felis* Fe/C-56	99.5	AE015925, *Chlamydia caviae* GPIC
cTF-068	*Chlamydia*	CP000051, *Chlamydia trachomatis* A/HAR-13	100	AE001273, *Chlamydia trachomatis* D/UW-3/CX
cTF-069	*Chlamydia*	GQ398031, *Chlamydia gallinacea* 08-1274	99	
cTF-074	*Parachlamydiaceae*	FR872580, *Parachlamydia acanthamoebae* UV-7	96.6	
cTF-088	*Chlamydia*	CP000051, *Chlamydia trachomatis* A/HAR-13	100	AE001273, *Chlamydia trachomatis* D/UW-3/CX
cTF-092	Unclassified *Chlamydiales*	JN616113, Uncultured bacterium	96.2	
**Children Control**				
cC-015	*Chlamydia*	AP006861, *Chlamydia felis* Fe/C-56	99.5	AE015925, *Chlamydia caviae* GPIC
cC-042	*Chlamydia*	AP006861, *Chlamydia felis* Fe/C-56	100	AE015925, *Chlamydia caviae* GPIC
cC-099	*Chlamydia*	AP006861, *Chlamydia felis* Fe/C-56	97.1	AE015925, *Chlamydia caviae* GPIC
**Adults Case**				
aTT-200	Unclassified *Chlamydiales*	AY114316, Uncultured *Chlamydia* sp.	94.6	
aTT-201	Unclassified *Chlamydiales*	JN606074, Chlamydiales bacterium NS11	95.1	
aTT-203	*Chlamydia*	AP006861, *Chlamydia felis* Fe/C-56	100	AE015925, *Chlamydia caviae* GPIC
aTT-207	Unclassified *Chlamydiales*	JN701140, Uncultured bacterium	90.2	
aTT-209	*Chlamydia*	AP006861, *Chlamydia felis* Fe/C-56	100	AE015925, *Chlamydia caviae* GPIC
aTT-211	Unclassified *Chlamydiales*	EU363464, Chlamydiales bacterium CRIB 32	98	
aTT-214	*Chlamydia*	AP006861, *Chlamydia felis* Fe/C-56	96.6	AE015925, *Chlamydia caviae* GPIC
aTT-216	Unclassified *Chlamydiales*	JN536584, Uncultured organism	93.6	
aTT-217	Unclassified *Chlamydiales*	JN606074, *Chlamydiales* bacterium NS11	98	
aTT-225	Unclassified *Chlamydiales*	JN701140, Uncultured bacterium	99	
aTT-226	Unclassified *Chlamydiales*	JX279901, Uncultured bacterium	91.2	
aTT-228	Unclassified *Chlamydiales*	HM063023, Uncultured bacterium	98	
aTT-237	*Chlamydia*	AP006861, *Chlamydia felis* Fe/C-56	99.5	AE015925, *Chlamydia caviae* GPIC
**Adults Control**				
aC-250	Unclassiefied *Chlamydiales*	HM063023, Uncultured bacterium	97.5	
aC-253	*Parachlamydia*	AM412760, *Parachlamydia acanthamoebae* OEW1	98.5	
aC-257	Unclassified *Chlamydiales*	AY114316, Uncultured *Chlamydia* sp.	91.2	
aC-259	*Chlamydia*	CP000051, *Chlamydia trachomatis* A/HAR-13	96.8	AE001273, *Chlamydia trachomatis* D/UW-3/CX
aC-260	Unclassified *Chlamydiales*	JX279901, Uncultured bacterium	97.5	
aC-261	*Chlamydia*	AP006861, *Chlamydia felis* Fe/C-56	98	AE015925, *Chlamydia caviae* GPIC
aC-262	Unclassified *Chlamydiales*	JN701140, Uncultured bacterium	95.8	

aC-263	Unclassified *Chlamydiales*	JN606074, *Chlamydiales* bacterium NS11	95.1	
aC-265	Unclassified *Chlamydiales*	AF364563, Uncultured *Chlamydiales* bacterium	95.5	
aC-268	Unclassified *Chlamydiales*	HM063023, Uncultured bacterium	98.5	
aC-279	Unclassified *Chlamydiales*	EU090709, Uncultured *Candidatus* Rhabdochlamydia sp.	87.7	
aC-282	Unclassified *Parachlamydiaceae*	AF364563, Uncultured *Chlamydiales* bacterium	96.1	
aC-284	*Chlamydia*	CP000051, *Chlamydia trachomatis* A/HAR-13	100	AE001273, *Chlamydia trachomatis* D/UW-3/CX
aC-285	Unclassified *Parachlamydiaceae*	LN995859.1 *Neochlamydia* sp. Trut23-12-2015	94.5	
aC-290	Unclassified *Chlamydiales*	JN536584, Uncultured organism	92.6	

Table S2. BLAST-n analysis of *Chlamydiae* 16S rRNA sequences detected in the ocular swabs of children and adults. Results are categorized based on the first and second BLAST-hit identification.

Household ID	Best BLAST match for 16S rRNA gene*	% Similarity	Second best BLAST match for 16S rRNA gene*	% Similarity
**Children Case**				
cTF-006	*Chlamydia trachomatis*	100	*Chlamydia trachomatis*	99
cTF-008	*Chlamydia trachomatis*	100	*Chlamydia trachomatis*	99
cTF-012	*Chlamydia trachomatis*	99	*Chlamydia trachomatis*	99
cTF-013	*Chlamydia trachomatis*	99	*Chlamydia trachomatis*	99
cTF-017	*Chlamydia trachomatis*	99	*Chlamydia trachomatis*	99
cTF-018	*Neochlamydia* SP.	95	*Neochlamydia endosymbiont of Acanthamoeba sp.*	95
cTF-020	*Chlamydia trachomatis*	100	*Chlamydia trachomatis*	99
cTF-021	*Chlamydia trachomatis*	100	*Chlamydia trachomatis*	99
cTF-025	*Chlamydiales* bacterium CRIB 32	95	*Parachlamydia acanthamoebae*	94
cTF-029	*Chlamydia trachomatis*	100	*Chlamydia trachomatis*	99
cTF-031	*Chlamydia trachomatis*	100	*Chlamydia trachomatis*	99
cTF-037	*Chlamydia trachomatis*	100	*Chlamydia trachomatis*	99
cTF-038	*Chlamydia trachomatis*	100	*Chlamydia trachomatis*	99
cTF-044	*Chlamydia trachomatis*	100	*Chlamydia trachomatis*	99
cTF-048	*Chlamydia trachomatis*	100	*Chlamydia trachomatis*	99
cTF-051	*Criblamydia sequanensis*	95	*Parachlamydia acanthamoebae*	93
cTF-057	*Chlamydia trachomatis*	100	*Chlamydia trachomatis*	100
cTF-059	*Chlamydia trachomatis*	100	*Chlamydia trachomatis*	98
cTF-060	*Chlamydia caviae/felis*	99	*Chlamydia felis*	99
cTF-068	100% *Chlamydia trachomatis*	100	*Chlamydia trachomatis*	99
cTF-069	*Chlamydia gallinacea*	99	*Chlamydia psittaci*	99
cTF-074	*Parachlamydia acanthamoebae*	97	*Parachlamydia acanthamoebae*	96
cTF-088	*Chlamydia trachomatis*	100	*Chlamydia trachomatis*	99
cTF-092	*Chlamydiales* bacterium CRIB33	92	*Criblamydiaceae* bacterium cvE70	90
**Children Control**				
cC-015	*Chlamydia caviae/felis*	99	*Chlamydia felis*	99
cC-042	*Chlamydia caviae/felis*	100	*Chlamydia felis*	100
cC-099	*Chlamydia* sp. 2742-308	98	*Chlamydia caviae*	97
**Adults Case**				
aTT-200	*Chlamydiales* bacterium NS11	95	*Chlamydiales* bacterium CRIB 32	94
aTT-201	*Chlamydiales* bacterium NS11	95	*Chlamydiales* bacterium CRIB 32	94
aTT-203	*Chlamydia caviae/felis*	100	*Chlamydia felis*	100
aTT-207	*Neochlamydia hartmannellae*	92	*Chlamydiales* bacterium NS13	92
aTT-209	*Chlamydia caviae/felis*	100	*Chlamydia felis*	100
aTT-211	*Chlamydiales* bacterium CRIB 32	98	*Chlamydiales* bacterium NS11	96
aTT-214	*Chlamydia* sp./ *Chlamydia pecorum*	97	*Chlamydia pecorum*	97
aTT-216	*Chlamydiales* bacterium CRIB 32	97	*Chlamydiales* bacterium NS11	94
aTT-217	*Chlamydiales* bacterium NS11	98	*Chlamydiales* bacterium CRIB 32	97
aTT-225	*Chlamydiales* bacterium NS11	95	*Chlamydiales* bacterium cvE18	94
aTT-226	*Chlamydiales* bacterium CRIB 32/ NS11	95	*Chlamydiales* bacterium CRIB 32	95
aTT-228	*Chlamydiales* bacterium NS11	96	*Chlamydiales* bacterium CRIB 32	95
aTT-237	*Chlamydia caviae/ felis*	99	*Chlamydia felis*	99
**Adults Control**				
aC-250	*Chlamydiales* bacterium CRIB 32	97	*Chlamydiales* bacterium NS11	95
aC-253	*Parachlamydia acanthamoebae*	98	*Parachlamydia acanthamoebae*	98
aC-257	*Simkania negevensis*	94	*Chlamydiales* bacterium NS11	93
aC-259	*Chlamydia trachomatis*	96	*Chlamydia trachomatis*	96
aC-260	*Chlamydiales* bacterium CRIB 32	97	*Chlamydiales* bacterium NS11	96
aC-261	*Chlamydia caviae/felis*	98	*Chlamydia felis*	98
aC-262	*Chlamydiales* bacterium CRIB 32	93	*Chlamydiales* bacterium NS11	92
aC-263	*Chlamydiales* bacterium NS11	97	*Chlamydiales* bacterium cvE18	94

aC-265	*Neochlamydia* sp.	95	*Candidatus Protochlamydia sp. cvE14*	94
aC-268	*Chlamydiales* bacterium CRIB 32	98	*Chlamydiales* bacterium NS11	96
aC-279	*Neochlamydia hartmannellae*	90	*Candidatus Clavichlamydia salmonicola*	89
aC-282	*Neochlamydia* sp.	94	*Candidatus Protochlamydia amoebophila*	94
aC-284	*Chlamydia trachomatis*	100	*Chlamydia trachomatis*	99
aC-285	*Protochlamydia naegleriophila*	96	*Protochlamydia naegleriophila*	95
aC-290	*Chlamydiales bacterium CRIB 32*	95	*Chlamydiales* bacterium NS11	94

* Uncultured bacteria were excluded from final BLAST hits by choosing the "Exclude" option in BLAST-n page for "Uncultured/environmental sample sequences".
